# Dysregulation of follicle fatty acid is a potential driver of human primary ovarian insufficiency

**DOI:** 10.1093/jmcb/mjaa044

**Published:** 2020-08-07

**Authors:** Lina Wang, Jihong Ma, Yixin Kang, Na Zhang, Xinyu Li, Hao Wang, Donghong Song, Mo Li, Huafang Gao, Xiumei Zhen

**Affiliations:** 1 Peking Union Medical College Graduate School, Beijing 100000, China; 2 National Research Institute for Health and Family Planning, Beijing 100000, China; 3 Reproductive Medicine Center of Henan Provincial People's Hospital, Zhengzhou 450003, China; 4 Center for Reproductive Medicine, Peking University Third Hospital, Beijing 100191, China; 5 National Clinical Research Center for Obstetrics and Gynecology, Beijing 100191, China; 6 Leslie Dan Faculty of Pharmacy, University of Toronto, Toronto M5S3M2, Canada

**Dear Editor**,

Primary ovarian insufficiency (POI) is defined as a significant reduction of the follicle pool and induction of amenorrhea before the age of 40 (Nelson, 2009), associated with a decreased level of estrogen and hypergonadotropic state in the blood. Since POI patients have few follicles, it is difficult to obtain human oocytes to decipher this disease. In mammalian ovary, granulosa cells establish direct communication with oocytes and modulate transcription and chromatin remodeling of the oocyte (De La Fuente and Eppig, 2001). However, the whole-genomic DNA methylation profile of human granulosa cells and the potential clinical insights are absent.

To profile the genomic methylation landscape, granulosa cells were harvested from three healthy donors and three representative POI patients (Supplementary Table S1). The sequencing parameters are reported in Supplementary Table S2. Genome-wide CpG island methylation level in all samples was generally similar. Of note, distinct differentially methylated regions (DMRs) and genes existed between the control and POI granulosa cells with the average length of 194 bp (Figure 1A;Supplementary Figure S1A–C). By Kyoto Encyclopedia of Genes and Genomes analysis, 20 pathways were potentially associated with POI (Supplementary Figure S1D). Among the genes involved in these pathways, 11 candidates that harbored high methylation levels in their promoters were identified in the POI group (Supplementary Table S3). Real-time PCR validated that 6 out of the 11 genes showed a substantial decrease in mRNA levels, with the mRNA level of *fatty acid-binding protein 3* (*FABP3*) decreased most (Figure 1B). FABPs are transporters of fatty acids that reversibly bind to and traffic fatty acids across cellular compartments (Hotamisligil and Bernlohr, 2015). However, the function of FABPs and related regulation of fatty acids in the ovary are largely unknown.

**Figure 1:**
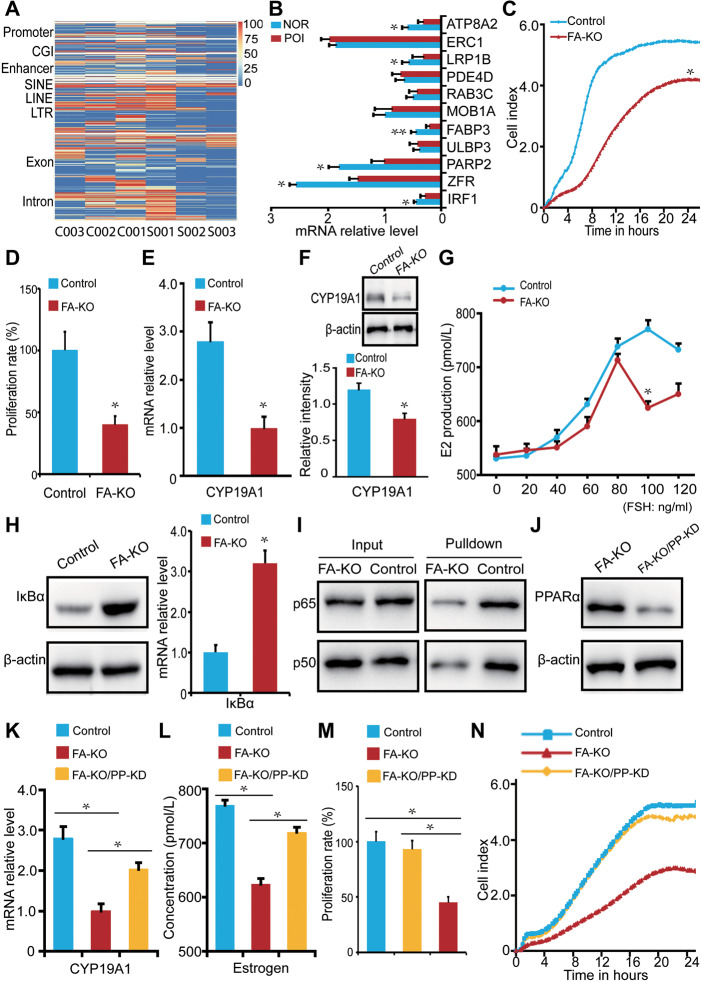
Molecular mechanism of fatty acid metabolism-related POI. (**A**) The methylation level of granulosa cell-specific DMRs (normal: C001–C003; POI: S001–S003). (**B**) The expression level of candidate genes in normal (NOR) and POI granulosa cells. For each gene, we analyzed each sample separately. (**C** and** D**) Cell proliferation in control and FA-KO cells. (**E** and** F**) mRNA and protein levels of CYP19A1 in control and FA-KO cells. (**G**) FSH-induced E2 production in control and FA-KO cells. (**H**) Protein level of IκBα in control and FA-KO cells. (**I**) The interaction between CYP19A1 and p65 or p50 in control and FA-KO cells. (**J**) The protein level of PPARα in FA-KO and FA-KO/PP-KD cells. (**K**–**N**) CYP19A1 mRNA expression (**K**), E2 production (**L**), and cell proliferation (**M** and** N**) in control, FA-KO, and FA-KO/PP-KD cells. *P <* *0.05; **P <* *0.01.

In both normal human primary granulosa cells and human granulosa cell line KGN, we examined the mRNA expression levels of all nine members in the FABP family. The mRNA expression levels of *FABP5* and *FABP6* were comparable to that of *FABP3*, whereas different from *FABP3*, their mRNA expression levels were not changed between the healthy donors and the POI patients, which ruled out potential effects of other FABP family members on POI (Supplementary Figure S2Aand B). Furthermore, *FABP3* was confirmed positively stained in granulosa cells throughout the development of ovarian follicles (Supplementary Figure S2C), implying a potential role of *FABP3* in granulosa cells.

To study the function of FABP3, we knocked it out in KGN cells (FA-KO) by the CRISPR/Cas9 system (Supplementary Figure S3Aand B). Using the xCELLigence RTCA system and MTT test, we found that the FA-KO cells exhibited a significantly reduced proliferation compared with the control (Figure 1C and D), consistent with the feature of POI (Nelson et al., 2013). It is well known that estrogen is essential for follicle development. CYP19A1 is responsible for the key rate-limiting catalysis of estrogens biosynthesis (Hsueh et al., 2015). Thus, we detected whether the loss of *FABP3* would affect CYP19A1. Interestingly, the expression of CYP19A1 was much lower in FA-KO cells (Figure 1E and F). Furthermore, under a gradient stimulation of follicle-stimulating hormone (FSH) from 0 to 120 ng/ml, the FA-KO cells responded less sensitively , and the total synthesis of E2 was accordingly reduced (Figure 1G). These data suggested that *FABP3* regulated E2 production by affecting the expression of CYP19A1 in granulosa cells.

To determine the underlying mechanism by which FABP3 affects CYP19A1 expression and thus regulates E2 synthesis in granulosa cells, we used DNA pull-down to screen potential transcription factors for CYP19A1. We therefore synthesized CYP19A1-PII double-stranded oligonucleotide, which dominantly functions in the ovary (Simpson et al., 1993), and incubated with nuclear protein extraction of granulosa cells. We found that 104 protein components existed in the pull-down complex analyzed by MS/MS (Supplementary Table S4). Of note, 18 unique peptides from p65 and 19 unique peptides from p50, the core proteins of the transcription factor NF-κB, were detected (Supplementary Figure S3C). Accumulative evidence has shown that PPARα could inhibit transcriptional activity of NF-κB by promoting the expression of the NF-κB inhibitor IκBα and thus decreasing the interaction between NF-κB and its targeted genes (Delerive et al., 2000). Meanwhile, PPARα could serve as a major transcriptional sensor of fatty acids (Poulsen et al., 2012). Thus, we hypothesized that FABP3 deficiency led to an increase of free fatty acid (FFA) that overactivated PPARα, which in turn suppressed the transcriptional activity of NF-κB on CYP19A1. As expected, the expression of IκBα in the FA-KO cells was significantly higher than that in the control (Figure 1H). Accordingly, CYP19A1-PII incubated with nuclear protein extracted from the FA-KO cells reduced the immune precipitation of p65 and p50 (Figure 1I). In the *FABP3* knockout/*PPARα* knockdown cells (FA-KO/PP-KD), the decreased PPARα recovered CYP19A1 expression, E2 production, and cell proliferation (Figure 1J–N). It is reported that PPARγ has a similar function to PPARα (Toda et al., 2003) and knockdown of *PPARγ* in FA-KO cells partially rescued CYP19A1 expression without significance. The expression of 17β-HSD type IV, an enzyme catalyzes E2 into the less activate estrone, was increased in FA-KO cells. Moreover, we ruled out the direct interaction between CYP19A1 and PPARα (Supplementary Figure S3D–F). Some key experiments were repeated by transient knockdown of *FABP3* (Supplementary Figure S4). These data suggested that the loss of FABP3 decreased CYP19A1 expression and E2 production via affecting NF-κB transcriptional activity.

It is known that physiological functions of FABPs depend on binding to FFA. In line with this, we found that the knockout of *FABP3* in granulosa cells resulted in an increase in FFA concentration compared with the control cells (Supplementary Figure S5A). To determine whether FFA level is related to POI in the clinic, we harvested follicular fluid from 19 healthy women and 10 POI patients (Supplementary Table S5). As expected, POI patients contained a higher level of FFA than the healthy controls (Supplementary Figure S5B). In particular, POI patients showed a significant disturbance of arachidonic acid metabolism in the follicular fluid (Supplementary Figure S5C). To validate the effect of FFA on the ovary, we cultured mouse ovaries *in vitro* and treated them with a commercial FFA mixture. After culturing for 48 h, the intracellular FFA in granulosa cells isolated from FFA-treated ovaries increased, accompanied with E2 reduction (Supplementary Figure S5Dand E). In addition, FFA treatment induced an increased expression and activity of PPARα, a decreased expression level of CYP19A1, and a lower proliferation rate as well as obvious apoptosis signals in granulosa cells isolated from the ovaries (Supplementary Figure S5F–K). These data suggested that extra FFA could be harmful to granulosa cells and the ovary.

Collectively, our data demonstrate that the dysregulation of follicle fatty acid is a potential driver of human POI, and the related factors such as FABP3 could be biomarkers for this disease.

*[Supplementary material is available at Journal of Molecular Cell Biology online. This work was supported by the National Key Research and Development Program of China (2016YFC1000604, 2017YFC1001501, and 2016YFC1000302) and the National Natural Science Foundation of China (81871160, 81672610, and 81521002). X.Z., H.G., and M.L. designed research; L.W., J.M., Y.K., N.Z., X.L., H.W., and D.S. performed research and analyzed data; X.Z., H.G., and M.L. wrote the paper; all authors approved the final version of the manuscript.]*

## Supplementary Material

mjaa044_Supplementary_Material
