# Supplementary material for: Dysregulation of follicle fatty acid is a potential driver of human primary ovarian insufficiency
Source: J Mol Cell Biol. 2020 Aug 7;12(10):817–9. doi: 10.1093/jmcb/mjaa044 (PMC7816672; doi:10.1093/jmcb/mjaa044)
Supplement: mjaa044_Supplementary_Material [file mjaa044_supplementary_material.pdf]

Supplementary Material

Dysregulation of follicle fatty acid is a potential driver of human primary ovarian insufficiency

Lina Wang[1,2,3,†] , Jihong Ma[4,5,†] , Yixin Kang[6], Na Zhang[4,5], Xinyu Li[4,5], Hao Wang[4], Donghong Song[4], Mo Li[4,5,*], Huafang Gao[2,*], Xiumei Zhen[4,5,*]

[1] Peking Union Medical College Graduate School, Beijing 100000, China

[2] National Research Institute for Health and Family Planning 100000, Beijing, China

[3] Reproductive Medicine Center of Henan Provincial People's Hospital, Zhengzhou 450003, Henan Province, China

[4] Center for Reproductive Medicine, Peking University Third Hospital, Beijing 100191, China

[5] National Clinical Research Center for Obstetrics and Gynecology, Beijing 100191, China

[6] Leslie Dan Faculty of Pharmacy, University of Toronto, Toronto M5S3M2, Canada

[*] Correspondence to: Mo Li, E-mail: limo@hsc.pku.edu.cn; Huafang Gao, E-mail: gaohuafang@nrifp.org.cn; or Xiumei Zhen: xmzhen1970@aliyun.com

[†] These authors contributed equally to this work

Contents

I. Materials and Methods 4

Clinic sample collection 4

Chemicals and antibodies 5

Methylated DNA analysis by whole-genome bisulfite sequencing 5

FABP3 knockout in KGN cells by CRISPR/Cas9 6

FABP3 knockdown in KGN cells by transient transfection 6

Cell index assay by xCELLigence technology 6

Cell proliferation assay by MTT test 7

E2 enzyme-linked immunosorbent assay 7

RNA isolation and real-time PCR 8

Biotin-streptavidin pull-down 9

Intracellular fatty acid analysis 9

Immunohistochemistry 10

Immunofluorescent staining 10

PPARα activity assay 11

Mouse ovary culture 11

Western blot 11

Statistical analysis 12

II. Supplementary Figures 13

Supplementary Figure S1. Whole genomic methylation landscape of POI ovarian granulosa cells. 13

Supplementary Figure S2. The expression of FABPs in granulosa cells and ovarian follicles. 15

Supplementary Figure S3. Function of FABP3 and related molecular mechanism. 17

Supplementary Figure S4. Key experiments involved in the function of FABP3 in POI were repeated by transient knockdown FABP3. 19

Supplementary Figure S5. Increase of FFA in FA-KO cells and in follicular fluid of POI. 21

III. Supplementary tables 22

Supplementary Table S1 Clinical samples information 22

Supplementary Table S2 Various sequencing parameters 23

Supplementary Table S3 DNA methylation level of candidate genes 24

Supplementary Table S4 Identification of related proteins by MS/MS 25

Supplementary Table S5 POI and NOR patients' clinic information 28

IV. Reference 29

I. Materials and Methods

Clinic sample collection

All human materials used in this study were received under the approval of the Institutional Review Board of Peking University Third Hospital(2016S2-007). Signed informed consent was obtained from all patients who participated in the study.

Human granulosa cells in follicular fluid were collected in the hospital from two groups of women: normal ovarian reserve patients with male factor infertility during assisted reproductive technology (ART) and POI patients. The diagnoses of POI were based on European Society of Human Reproduction and Embryology 2016 diagnostic criteria (European Society for Human et al., 2016). Women with histories of anticancer treatment, pelvic surgery, ovarian infection, and/or autoimmune disease were excluded from the study. Granulosa cells were harvested from each follicular fluid sample according to the method as previously described (Lobb and Younglai, 2006). Briefly, follicular fluid samples were placed on Ficoll-paque gradient and centrifuged for 20 min at 900 *g* at room temperature. The interphase that mainly contains ovarian granulosa cells was collected and washed in Dulbecco's phosphate-buffered saline (DPBS). Then, 0.02 mg/ml collagenase was added and incubated for 4 minutes to disperse cell clumps. After further washing with DPBS, the samples were added with CD45-labeled Dynabeads to deplete leukocyte contaminations.

For follicular fluid fatty acid analysis, follicular fluid was obtained from women treated with ART at the hospital. The ovarian functions were recorded from women with POI and healthy controls. Follicular fluids were collected in capped disposable polypropylene tubes and centrifuged at 4750 RPM for 10 min. The supernatants were collected for liquid chromatography tandem mass spectrometry (LC/MS/MS). Thermo Scientific™ Dionex™ UltiMate™ 3000 Rapid Separation LC (RSLC) system (Thermo Fisher Scientific,

Waltham, MA, USA) was used to perform fatty acid quantification. The human metabolome database and lipid maps were identified. All data were processed using Progenesis QI data analysis software (Nonlinear Dynamics, Newcastle, UK).

Chemicals and antibodies

All chemicals and reagents were purchased from Sigma (St. Louis, MO, USA), except for those specifically mentioned: streptavidin magnetic beads (Thermo Fisher Scientific, Waltham, MA, USA); anti-FABP3 antibody (Abcam, Cambridge, UK; Cat# ab45966), IF (1:200), IHC (1:1000), WB (1:300); IκBα antibody (Abcam, Cambridge, UK; Cat# ab32518), WB (1:1000); anti–NF-κB p65 antibody (Abcam, Cambridge, UK; Cat# ab16502), WB (1:2000); anti-PPAR alpha antibody (Abcam, Cambridge, UK; Cat# ab24509), WB (1:100); CYP19 antibody (Santa Cruz Biotechnology, Dallas, TX, USA; Cat# sc-374176), WB (1:200); MagniSort™ Human CD45 Depletion Kit (Invitrogen, Carlsbad, CA, USA; Cat# 8804-6802-74); mouse anti-human CD45 (BD Pharmingen™, San Jose, CA, USA; Cat# 555482).

Methylated DNA analysis by whole-genome bisulfite sequencing

Genomic DNA was extracted from purified granulosa cells. DNA purity was checked using the NanoPhotometer® spectrophotometer (IMPLEN, CA, USA). DNA concentration was measured using the Qubit® DNA Assay Kit in the Qubit® 2.0 Flurometer (Life Technologies, CA, USA). A total of 5.2 μg genomic DNA spiked with 26 ng lambda DNA was fragmented by sonication to 200 to 300 bp with Covaris S220, followed by end repair and adenylation. Cytosine-methylated barcodes were ligated to sonicated DNA as per the manufacturer's instructions. Then, these DNA fragments were treated twice with bisulfite using the EZ DNA Methylation-GoldTM Kit (Zymo Research), before the resulting single-strand DNA fragments were amplified by PCR using KAPA HiFi HotStart Uracil + ReadyMix (2X). Library preparations were sequenced on an

Illumina Hiseq 2500 at Annoroad Gene Technology Co., Ltd (Beijing, China), and 150-bp paired-end reads were generated. Image analysis and base calling were performed with the Illumina CASAVA pipeline, and 150-bp paired-end reads were finally generated.

FABP3 knockout in KGN cells by CRISPR/Cas9

The sgRNAs for *FABP3* gene CRISPR were designed using the website http://crispr.mit.edu/. Two sgRNAs with the lowest off-target scores were selected. Their sequences are Oligo1: 5'-CACCGCTTGCTGTCCACTAGCTTCC-3', Oligo2: 5'-AAACGGAAGCTAGTGGACAGCAAGC-3'. The sgRNAs were cloned into the lentiCRISPR transfer plasmid for virus production in HEK293T cells. KGN cells were infected with the harvested viral. Viral produced from the empty lentiCRISPR transfer plasmid in HEK293T cells was used as control viral. After infection, KGN cells were selected with 0.5 μg/ml puromycin for 3 to 4 weeks. *FABP3* knockout cells from different clones were further selected by western blot with anti-FABP3 antibody.

FABP3 knockdown in KGN cells by transient transfection

Targeted small interfering RNAs (siRNAs) were transfected into KGN cells using Lipofectamine RNAiMAX reagent (Invitrogen) according to the manufacturer's instructions. After transfection for 2 days, the knockdown efficiency was confirmed by quantitative real-time PCR and Western blot.

Cell index assay by xCELLigence technology

Cell index was assessed using the xCELLigence technology (Acea Bioscience, San Diego, CA, USA, distributed by Roche Diagnostics), which allowed long-term monitoring of live cells in a noninvasive manner (Al Nakouzi et al., 2016). In brief, 10,000 KGN cells were seeded in each well of E-16-well plates (Roche).

Cell index was monitored for 24 hours at 37°C in the incubator. Microelectrodes on the bottom of the plates were used to detect impedance changes proportional to the number of adherent cells. The impedance value of each well was automatically monitored by Real-Time Cell Analyzer (RTCA) software and recorded. These experiments were conducted in triplicate and repeated twice.

Cell proliferation assay by MTT test

Discard media from cell cultures. The adherent cells were measured by adding 50 μL of serum-free media and 50 μL of MTT solution into each well. Incubate the plate at 37°C for 3 hours. After incubation, add 150 μL of MTT solvent into each well. Absorbance was measured at 570 nm using a multi-well spectrophotometer (Bio-Rad, Hercules, CA, USA).

E2 enzyme-linked immunosorbent assay

E2 concentrations in culture supernatants were determined using the Estradiol Human ELISA Kit (Thermo Fisher Scientific, Waltham, MA, USA) following the manufacturer's standard protocol. Control KGN cells and *FABP3* knockout cells were treated with different concentrations of follicle-stimulating hormone (FSH; 0, 20, 40, 60, 80, 100, 120 ng/ml), respectively. Mediums were collected after 48 hours and stored at –80°C for subsequent analysis. Estrogen standards (provided in the kit) and cultured supernatants were placed into 96 wells, respectively, mixed with estradiol–horseradish peroxidase (HRP) conjugate and anti-estradiol, and incubated for 2 hours at room temperature on a horizontal shaker set at 700 rpm. The solutions were thoroughly aspirated, and the wells were washed extensively. Afterward, chromogen solution was added and incubated for 30 min at room temperature in the dark. After addition of stop solution, the absorbance of each well was measured at 450 nm by a spectrophotometer (BioRad, Hercules, CA, USA). Estrogen concentrations were calculated from the standard curves.

RNA isolation and real-time PCR

Total RNA from granulosa cells was extracted using Trizol reagent (Invitrogen). Then, the RNA was reverse-transcribed using RevertAid First Strand cDNA Synthesis Kit (Thermo Scientific). Real-time PCR analysis was performed using SYBR Green PCR master mix with the QuantStudio 3 Real-Time PCR system (Applied Biosystems, Singapore). The primer sequences are IRF1, 5'-ATGCCCATCACTCGGATGC-3' (sense), 5'-CCCTGCTTTG TATCGGCCTG-3' (anti-sense); ZFR, 5'-AATGGCGACC GGCAACTAC-3' (sense), 5'-TGAGAATAGGCTACACCCGAAG-3' (anti-sense); PARP2, 5'-GCCTTGCTGT TAAAGGGCAAA-3' (sense), 5'-TCCTTCACAATACA CATGAGCC-3'(anti-sense); ULBP3, 5'-TCTATGGGTCACCTAGAAGAGC-3' (sense), 5'-TCCACTGGGTGTG AAATCCTC-3' (anti-sense); FABP3, 5'-TCGAGATGGAGAGTGAGAAGAA-3' (sense), 5'-TCCGTGACGATCTTGAAGTTG-3' (anti-sense); MOB1A, 5'-CAGC AGCCGCTCTTCTAAAAC-3' (sense), 5'-CCTCAGGCAACATAACAGCTTG-3' (anti-sense); RAB3C, 5'-ATCATCGGCAATAGCAGTGTG-3'(sense), 5'-AGGCTG TGGTGATAGTCCTGT-3' (anti-sense); PDE4D, 5'-GACCAATGTCTCAGATCAG TGG-3' (sense), 5'-GTCAAGGGCCGGTTACCA G-3'(anti-sense); LRP1B, 5'-TTT CTCCTCGCCTTACTCACT-3' (sense), 5'-CACACAACTGCTGATCTCGGT-3' (anti-sense); ERC1, 5'-TGGCATGGCCAAACCTAA -3' (sense), 5'-AGGTGTTTCT GAACCCACATTTT-3' (anti-sense); ATP8A2, 5'-CCCGCACCATTTACCTCA AC-3' (sense), 5'-GGCACCAGGGTGGTATATCTTC-3' (anti-sense); CYP19A1, 5'-GGTGACCTGACAAGAGAGAATG-3'(sense), 5'-GGGTGCTTTGCAATGAGAAATAG-3'(anti-sense); IκBα, 5'-GCAAAATCCTGACCTGGTGT-3'(sense), 5'-GCTCGTCCTCTGTGAACTCC-3'(anti-sense); PPARα, 5'-AGGGCCTCCCTCCTACGCTTG-3'(sense), 5'-GGGTGGCAGGAAGGGAACAGA-3'(anti-sense). The primer sequences for reference gene ACTB are: 5'-TGCCCATCTACGAGGGGTAT-3'(sense), 5'- CTTAATGTCACGCACGATTTCC-3'(anti-sense). The next

cycling conditions were preincubation at 95°C for 2 min, followed by 40 cycles of denaturation at 95°C for 15 sec, and annealing/extension at 60°C for 1 min. The results were analyzed by the relative quantification ($\Delta\Delta C_T$) method.

Biotin-streptavidin pull-down

CYP19A1 promoter II (PII), the promoter most proximal to exon II of the gene, was amplified by PCR using 5'-biotin–labeled primer. The primer sequences for CYP19A1 PII are 5'-CACTGAAAATGCATTTAATGATGA-3' (sense), 5'-CAGATAATTGGT CTGAAAATTTTC-3' (anti-sense). The 5'-biotinylated DNA of CYP19A1 PII was immobilized to streptavidin beads following the manufacturer's protocol. The 5'-biotinylated DNA beads were incubated with proteins lysed from KGN cells for 4 hours at 4°C on a rotating shaker. The beads were then washed three times with cold PBS. After the last wash, the beads were resuspended in protein loading buffer, and the proteins were liberated from the beads by boiling for 5 min. The harvested proteins were subjected to sodium dodecyl sulfate–polyacrylamide gel electrophoresis (SDS-PAGE) and tandem mass spectrometry (MS/MS) analysis.

Intracellular fatty acid analysis

Intracellular fatty acids were measured with the Free Fatty Acid Quantitation Kit (Sigma, St. Louis, MO, USA) according to the manufacturer's standard protocol. In brief, control KGN cells and *FABP3* knockout cells were treated with 100 ng/mL FSH for 48 hours followed by PBS washes and cell harvest. Cellular lipids were extracted by a chloroform-Triton X mixture at a ratio of 99:1(v/v) and centrifuged at 13,000 *g* for 10 min to achieve phase separation. The organic phase was dried under the air, and the lipids were solubilized in 200 μl free fatty acid (FFA) buffer and mixed for 5 min. Palmitic acid standard (provided in the kit) and culture supernatants were placed into 96 wells, respectively. Two microliters of ACS reagent was added into each well

and incubated for 30 min at 37°C. Next, 50 µl Master Reaction Mix (including fatty acid probe and enzyme mix) was mixed well and incubated for 30 min at 37°C in dark. Finally, the absorbance of each well was measured at 570 nm on a spectrophotometer. Concentrations of the samples were calculated according to the standard curve.

Immunohistochemistry

Human ovary specimens were fixed overnight in 10% neutral buffered formalin, embedded in paraffin, and sectioned. Embedding and sectioning were performed by the Immunohistochemistry Core at Peking University Third Hospital. Sections were then deparaffinized in xylene. Endogenous peroxidase activity of the sections was blocked in 3% H_2O_2 solution in methanol at room temperature. Then, the slides were incubated with FABP3 antibody overnight at 4°C. After washing three times in PBS, the sections were incubated with HRP-conjugated secondary antibody for 30 min at room temperature. Finally, the sections were observed under microscopy.

Immunofluorescent staining

KGN cells were fixed with 4% paraformaldehyde in PBS (pH 7.4) for 30 min and were permeabilized with 0.5% Triton X-100 in PBS for 20 min at room temperature. After blocking in 1% bovine serum albumin (BSA)–supplemented PBS for 1 hour at room temperature, the cells were incubated with anti-FABP3 antibody overnight at 4°C. After washing three times with PBS containing 0.1% Tween-20 (TBST) and 0.01% Triton X-100, cells was incubated with appropriate fluorescent antibodies for 1 hour at room temperature. After further washing, cytoplasmic actin was counterstained with Alexa Fluor® 555 conjugated phalloidin for 1 hour at room temperature, and nuclei were counterstained with Hoechst 33342 (10 µg/mL) for 15 min. Finally, the cells were mounted on glass slides and observed under a confocal laser scanning microscope at 63/1.40× (Carl Zeiss 710).

PPARα activity assay

Nuclear extracts were isolated from the granulosa cells of FFA-treated mouse ovaries and POI patients using NE-PE Nuclear and Cytoplasmic Extraction Reagent Kit (Thermo Fisher, Pittsburgh, USA, Ca#78833,). PPARα activity was assessed using the PPAR (α, δ, γ) Transcription Factor Assay Kit (Abcam, Toronto, Canada, Ca#ab133113) following the manufacturers' instructions.

Mouse ovary culture

Six-week-old female ICR mice were obtained from Peking University Department of Medicine Animal Center. All animals were housed under a 12-hour light/12-hour dark regime at 22-24°C. The mice were sacrificed by cervical dislocation, and their ovaries were dissected free of fat and mesentery. The culture of ovaries was performed as previously described (Morgan et al., 2015) with α-minimal essential media supplemented with 3 mg/ml BSA, 100 IU/ml penicillin, and 50 μg/ml streptomycin. Culture medium and culture plates were equilibrated for at least 1 hour in a 37°C, 5% CO_2 incubator before use. After being culled from mice, the ovaries were placed on the polycarbonate membrane in the culture medium in the absence or presence of different concentrations of FFA (0, 100, or 300 μM). Forty-eight hours later, the media were collected for the measurement of E2 level by enzyme-linked immunosorbent assay.

Western blot

Cells were lysed in 4x LDS sample buffer (Thermo Fisher Scientific, Waltham, MA, USA) containing protease inhibitor and boiled in water for 5 min. The proteins were separated on SDS-PAGE and electrically transferred onto polyvinylidene fluoride membranes (Millipore, Darmstadt, Germany). After blocking in TBST containing 5% skimmed milk for 2 hours at room temperature, the membranes were incubated with indicated primary

antibodies overnight at 4°C. After washing in TBST, the membranes were incubated for 1 hour at 37°C with 1:1,000 dilution of HRP-conjugated secondary antibody. Finally, protein bands were visualized by an enhanced chemiluminescence detection system (BioRad, Hercules, CA, USA).

Statistical analysis

All experiments were performed at least three times. Comparison between two groups were analyzed using Student's t-test, and comparison among three or more groups were analyzed using one-way ANOVA or ANOVA for repeated measurement design data. Data were expressed as mean ± SD, P values less than 0.05 were considered statistically significant.

II. Supplementary Figures

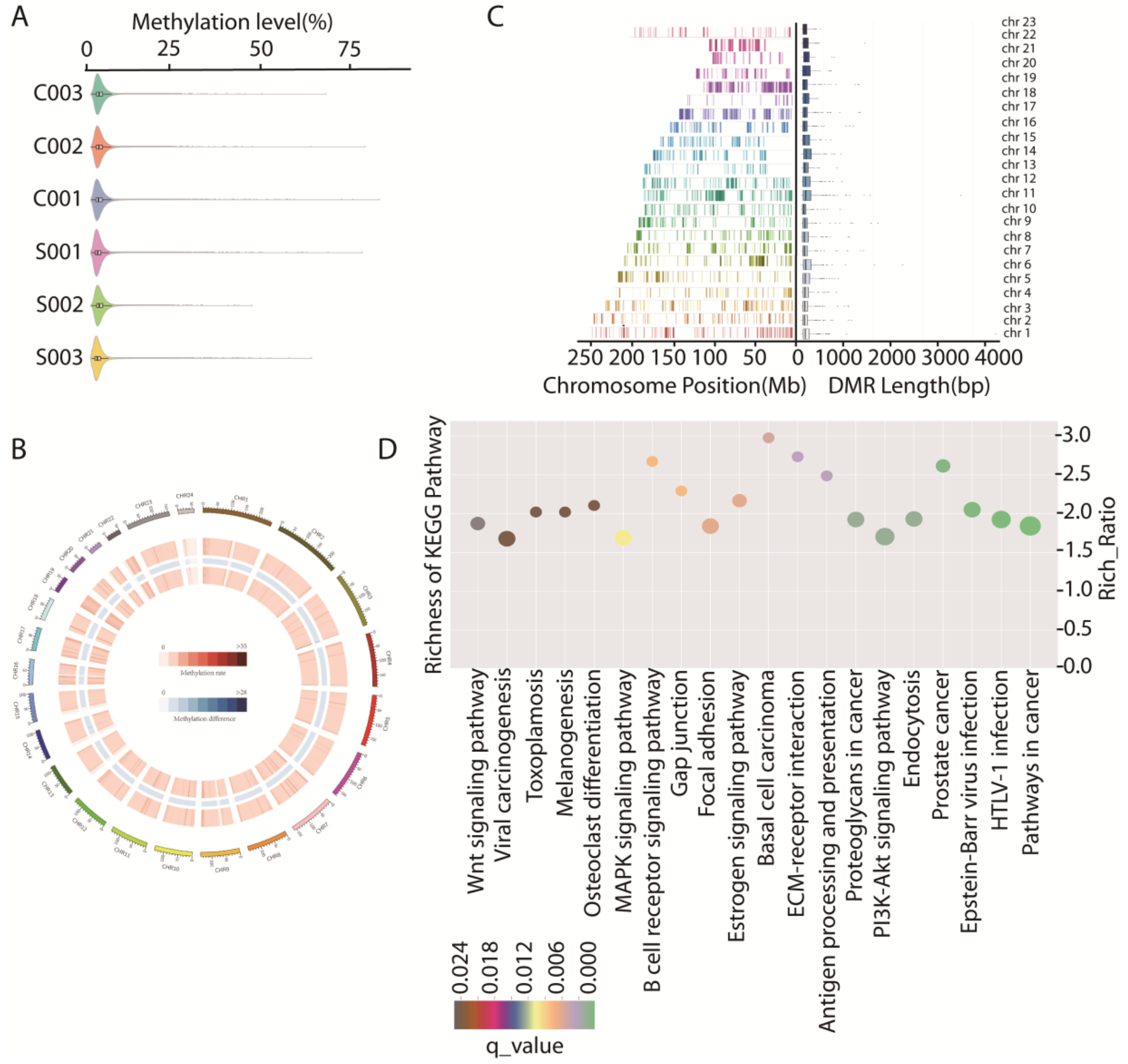

Supplementary Figure S1. Whole genomic methylation landscape of POI ovarian granulosa cells.

(A) Whole genome methylation level in granulosa cells of normal women (C001, C002, C003) and POI (S001, S002, S003). Each violin represents an individual sample. **(B)** Circular plot for genome-wide DNA methylation signals in different chromosomes of granulosa cells. Inner-most circle in red indicates the methylation

percentage of POI. Outer circle in red indicates that of NOR. Blue circle indicates the percentage of methylation difference between POI and NOR. **(C)** Distribution and length of granulosa cells-specific DMRs. **(D)** KEGG pathways enriched among the differentially methylated genes. Rich ratio is defined as the percentage of differentially methylated genes annotated in one pathway term divided by the expected percentage of that. The greater the rich ratio the higher the enrichment. Q-value is corrected P-value ranging from 0~1, with a lower value means greater intensiveness. The size of the point represents the number of selected genes in the pathway**.**

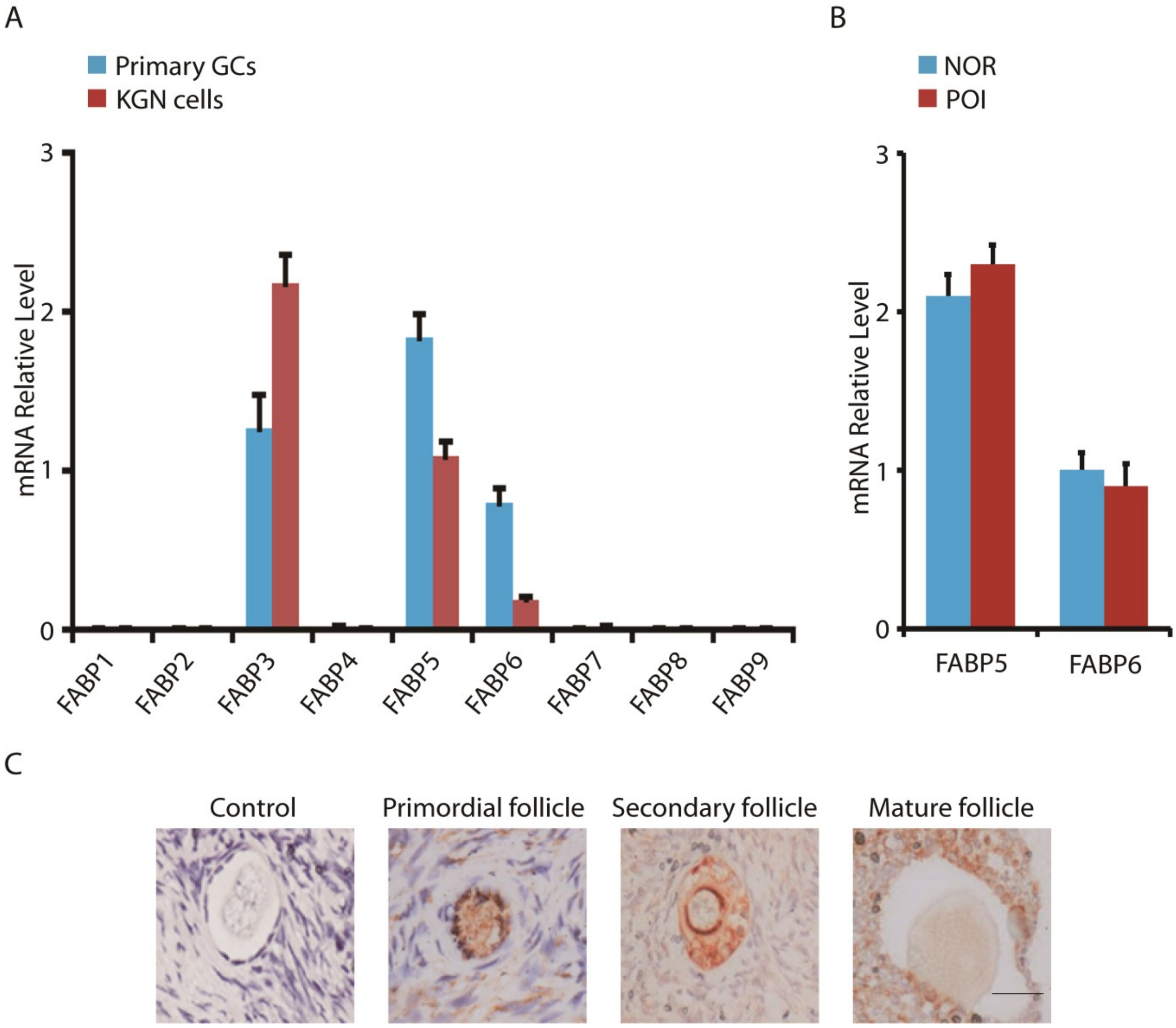

Supplementary Figure S2. The expression of FABPs in granulosa cells and ovarian follicles.

(A) The mRNA expression levels of *FABP1-9* genes in primary granulosa cells (primary GCs) and KGN cells. The expression values were summarized from three independent repeats. **(B)** The expression of FABP5 and FABP6 in granulosa cells of normal women and POI. **(C)** Immunohistochemical staining of FABP3 in human ovarian follicles. FABP3 is expressed throughout the development of ovarian follicles. IgG staining was used as the negative control. Scale bar, 50μm.

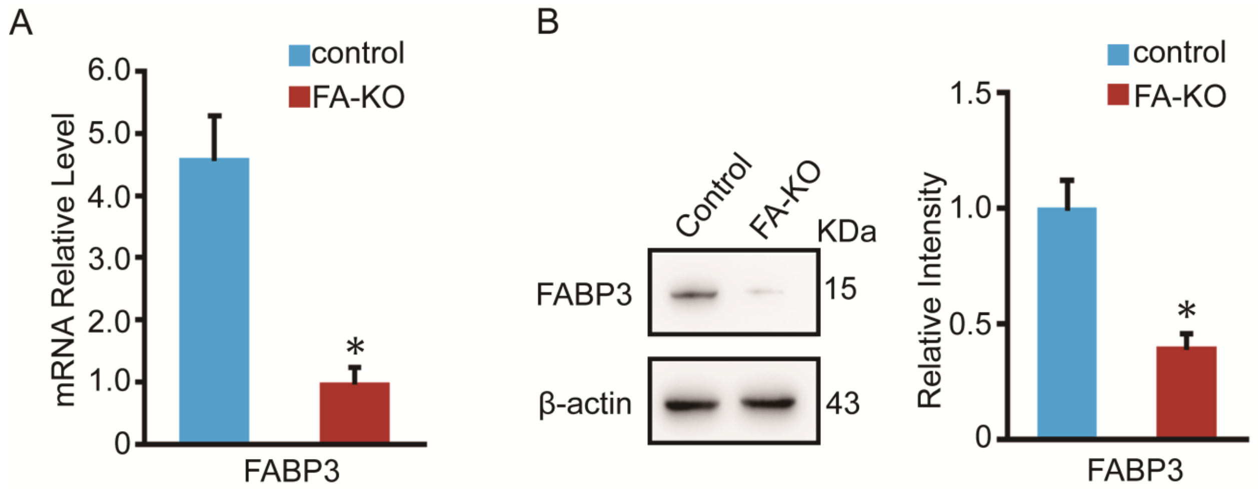

C

Related proteins identification by MS/MS
(see full information in Supplementary table S4)

Asscession	Description	Area	Peptides
NP_001138610.1	transcription factor p65 isoform 2	9.51E+08	18
NP_001349801.1	peroxisome proliferator-activated receptor alpha	5.09E+08	18
NP_001158884.1	nuclear factor NF-kappa-B p105 subunit isoform 2 proprotein	2.90E+08	19
NP_065390.1	NF-kappa-B inhibitor alpha	2.20E+08	13

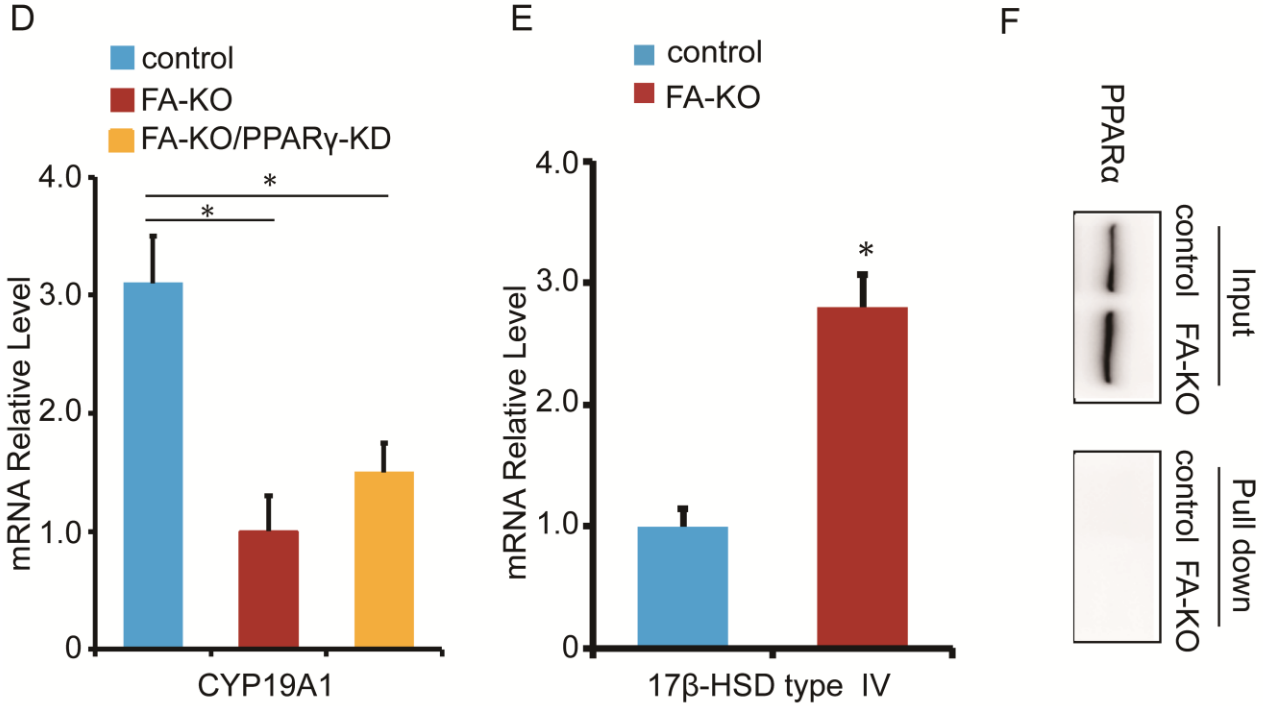

Supplementary Figure S3. Function of FABP3 and related molecular mechanism.

(A) mRNA expression level of *FABP3* in control and FA-KO cells. **(B)** Protein level of FABP3 in control and FA-KO cells. Relative intensity of the bands was summarized in the histogram. **(C)** Identification of captured proteins by CYP19A1 PII pull-down through MS/MS. **(D)** CYP19A1 mRNA expression in control, FA-KO and FA-KO/PP-KD cells. **(E)** 17β-HSD type IV mRNA expression in control and FA-KO cells. **(F)** The interaction between CYP19A1 and PPARα in control and FA-KO cells. *, P<0.05.

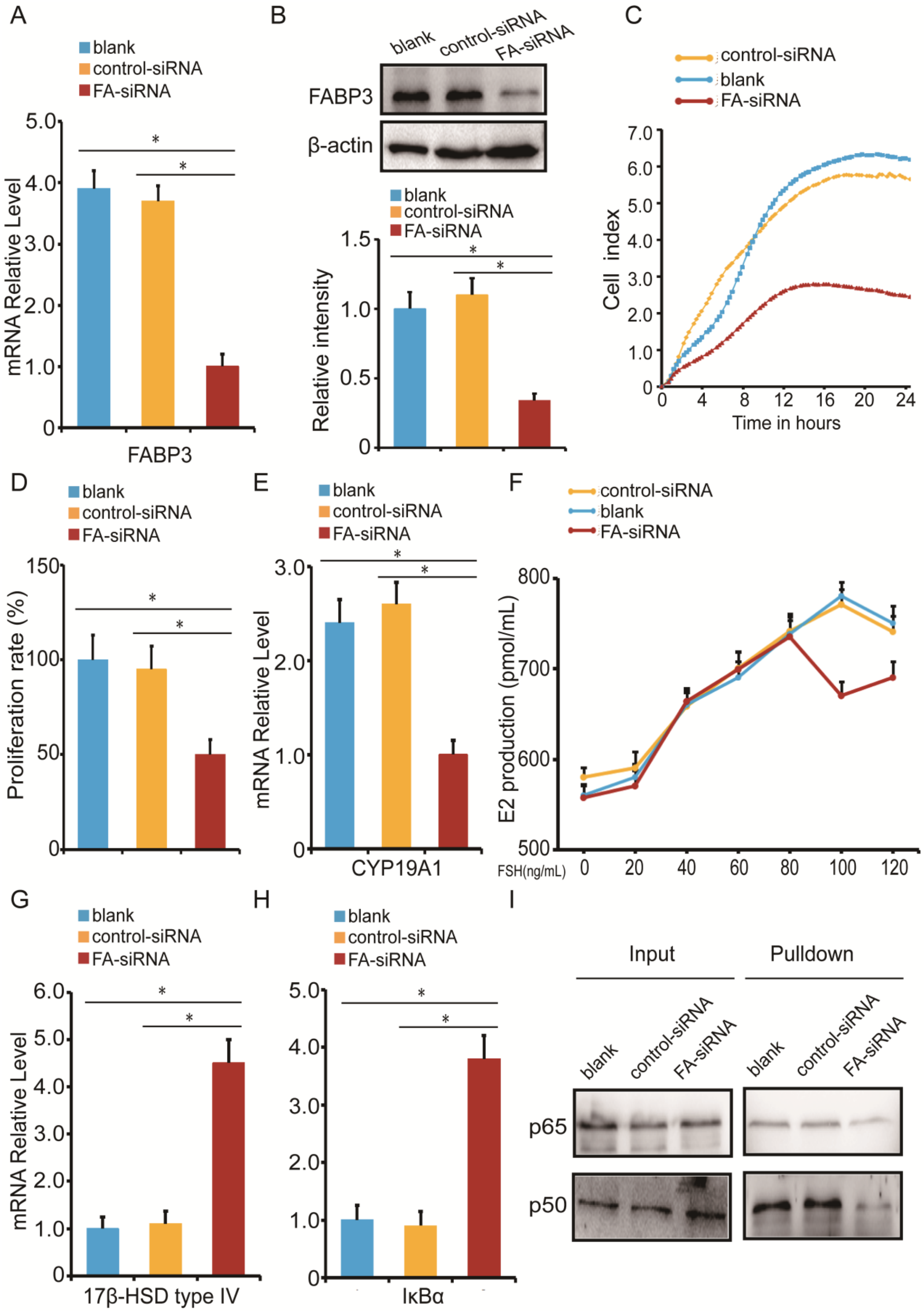
A
blank
control-siRNA
FA-siRNA
mRNA Relative Level
5.0
4.0
3.0
2.0
1.0
0
*
*
FABP3
B
blank
control-siRNA
FA-siRNA
FABP3
β-actin
blank
control-siRNA
FA-siRNA
Relative intensity
1.5
1.0
0.5
0
*
*
C
control-siRNA
blank
FA-siRNA
Cell index
7.0
6.0
5.0
4.0
3.0
2.0
1.0
0
0
4
8
12
16
20
24
Time in hours
D
blank
control-siRNA
FA-siRNA
Proliferation rate (%)
150
100
50
0
*
*
E
blank
control-siRNA
FA-siRNA
mRNA Relative Level
3.0
2.0
1.0
0
*
*
CYP19A1
F
control-siRNA
blank
FA-siRNA
E2 production (pmol/mL)
800
700
600
500
FSH(ng/mL)
0
20
40
60
80
100
120
G
blank
control-siRNA
FA-siRNA
mRNA Relative Level
6.0
5.0
4.0
3.0
2.0
1.0
0
*
*
17β-HSD type IV
H
blank
control-siRNA
FA-siRNA
5.0
4.0
3.0
2.0
1.0
0
*
*
IκBα
I
Input
Pulldown
blank
control-siRNA
FA-siRNA
blank
control-siRNA
FA-siRNA
p65
p50

Supplementary Figure S4. Key experiments involved in the function of FABP3 in POI were repeated by transient knockdown FABP3.

(A) mRNA expression levels of *FABP3* in blank, control-siRNA and FA-siRNA transfected cells. **(B)** Protein levels of FABP3 in blank, control-siRNA and FA-siRNA transfected cells. **(C-I)** Cell proliferation **(C-D)**, CYP19A1 mRNA expression **(E)**, E2 production **(F)**, 17β-HSD type IV mRNA expression **(G)**, IκBα mRNA expression **(H)** and the interaction between CYP19A1 and p65 or p50 **(I)** in blank, control-siRNA and FA-siRNA transfected cells. *, $P<0.05$.

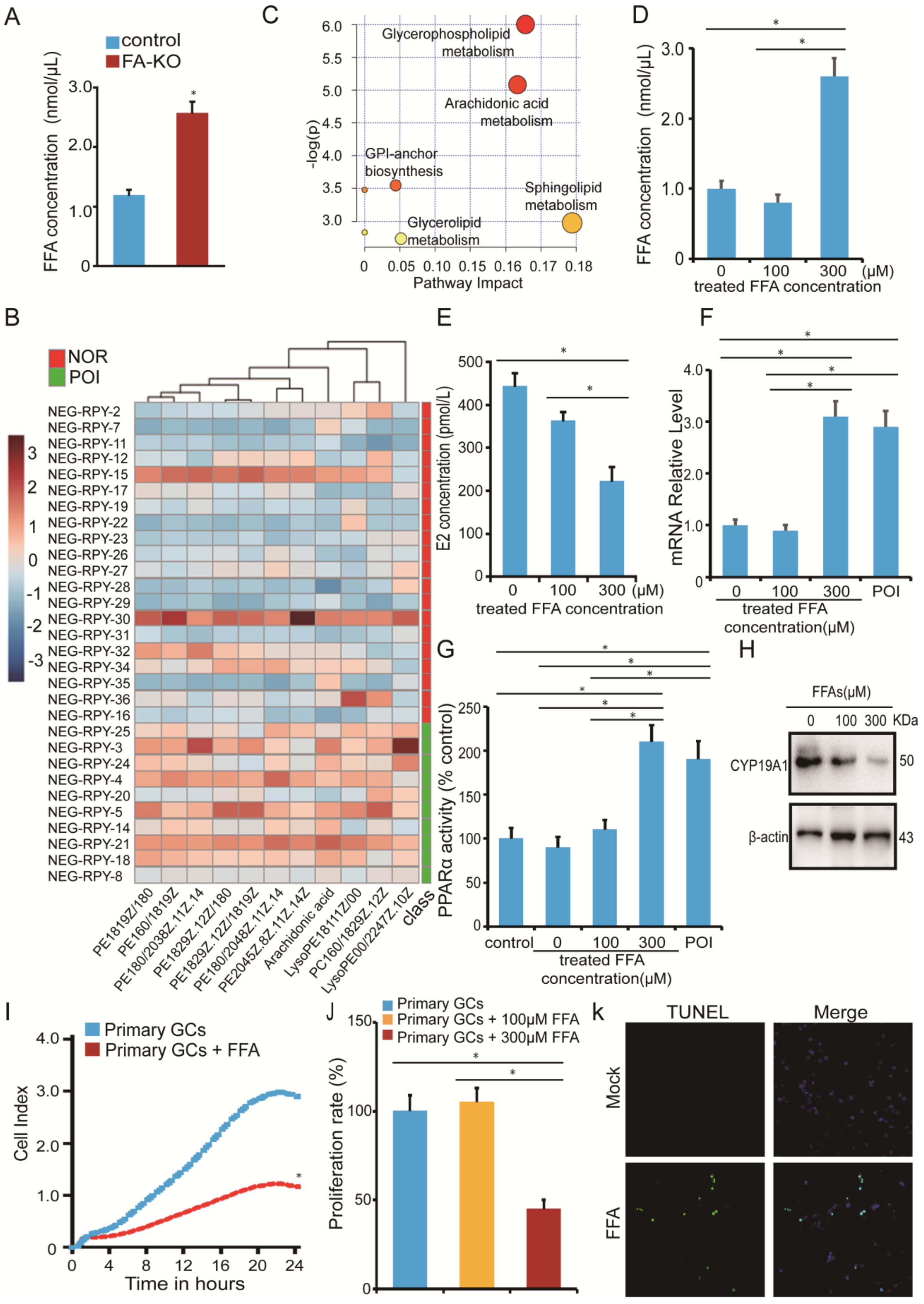
A
control
FA-KO
FFA concentration (nmol/μL)
3.0
2.0
1.0
0
*
C
Glycerophospholipid metabolism
Arachidonic acid metabolism
GPI-anchor biosynthesis
Sphingolipid metabolism
Glycerolipid metabolism
-log(p)
Pathway Impact
D
FFA concentration (nmol/μL)
0
100
300
(μM)
treated FFA concentration
B
NOR
POI
NEG-RPY-2
NEG-RPY-7
NEG-RPY-11
NEG-RPY-12
NEG-RPY-15
NEG-RPY-17
NEG-RPY-19
NEG-RPY-22
NEG-RPY-23
NEG-RPY-26
NEG-RPY-27
NEG-RPY-28
NEG-RPY-29
NEG-RPY-30
NEG-RPY-31
NEG-RPY-32
NEG-RPY-34
NEG-RPY-35
NEG-RPY-36
NEG-RPY-16
NEG-RPY-25
NEG-RPY-3
NEG-RPY-24
NEG-RPY-4
NEG-RPY-20
NEG-RPY-5
NEG-RPY-14
NEG-RPY-21
NEG-RPY-18
NEG-RPY-8
PE1819Z/180
PE160/1819Z
PE180/2038Z.11Z.14
PE1829Z.12Z/180
PE1829Z.12Z/1819Z
PE180/2048Z.11Z.14
PE2045Z.8Z.11Z.14Z
Arachidonic acid
LysoPE1811Z/00
PC160/1829Z.12Z
LysoPE00/2247Z.10Z
class
E
E2 concentration (pmol/L)
treated FFA concentration
F
mRNA Relative Level
POI
treated FFA concentration(μM)
G
PPARα activity (% control)
control
treated FFA concentration(μM)
H
FFAs(μM)
KDa
CYP19A1
β-actin
50
43
I
Primary GCs
Primary GCs + FFA
Cell Index
Time in hours
J
Primary GCs
Primary GCs + 100μM FFA
Primary GCs + 300μM FFA
Proliferation rate (%)
k
TUNEL
Merge
Mock
FFA

Supplementary Figure S5. Increase of FFA in FA-KO cells and in follicular fluid of POI.

(A) The concentration of FFA in control and FA-KO cells. **(B)** Clustering analysis of FFAs in normal and POI follicular fluid (MetaboAnalyst 3.0). The color of each sections is proportional to the significance of the change in metabolites (red, up-regulated; blue, down-regulated). Follicular fluid fatty acid of 10 POI patients and 19 healthy women were analyzed separately, and showed similar expression trend. (**C)** Pathway impact analysis of 29 samples by MetaboAnalyst 3.0. Metabolic pathways with value >0.05 were considered to be perturbed. **(D)** FFA concentration in granulosa cells isolated from mouse ovaries treated with FFA. **(E)** E2 synthesis in cultured mouse ovaries treated with FFA. **(F-G)** mRNA expression levels **(F)** and activity **(G)** of PPARα in granulosa cells isolated from mouse ovaries treated with FFA and from POI patients. (**H)** Protein level of CYP19A1 in cultured ovarian treated with FFA. (**I-J)** The proliferation of granulosa cells from mouse ovarian treated with FFA. (**K)** TUNEL staining of granulosa cells isolated from mouse ovary treated with or without FFA. Scale bar, 40 μm. *, $P<0.05$.

III. Supplementary tables

Supplementary Table S1 Clinical samples information

Supplementary Table S1 Clinical samples information

Groups	Number	Age	Systolic Pressure (mmHg)	Diastolic Pressure (mmHg)	BMI	FSH(IU/L)	LH (IU/L)	PRL (ng/ml)	T (ng/dl)	E2 (pg/ml)	TSH (uIU/ml)	Ultrasonography (numbers of unilateral ovaries)
NOR	C001	27	115	72	19.10	7.12	5.45	10.13	20.93	76.3	1.74	8 follicles
	C002	28	120	80	23.18	6.28	4.2	6.22	27.65	79.3	2.5	6 follicles
	C003	28	119	80	20.90	7.66	3.47	7.59	25.52	65.5	1.65	7 follicles
POI	S001	28	117	64	21.48	27.39	8.87	11.5	28.91	11.54	2	1 follicles
	S002	29	115	70	21.91	26.19	9.63	8.76	22.49	15.2	1.56	1 follicles
	S003	25	112	80	22.56	28.61	7.68	23.66	18.76	18.7	2.01	1 follicles

Supplementary Table S2 Various sequencing parameters

Supplemental Table S2 Various sequencing parameters

Sample	Raw Reads	Mapped Reads	Mapping Efficiency (%)	Bisulfite Conversion Efficiency (%)	Depth (X/per strand)	Unique Mapped Ratio (%)	Insert Size (bp)	Clean Q30 Bases Rate (%)
C001	936,184,752	670,737,792	91.3	99.7	14.35	86.9	281.72	93.88
C002	875,988,556	617,649,628	92.5	99.7	13.21	88.0	268.32	93.06
C003	867,579,210	624,896,780	92.1	99.7	13.35	87.6	279.10	94.24
S001	898,167,114	601,768,558	92.7	99.7	12.78	87.6	255.41	92.36
S002	718,595,990	586,902,810	84.8	99.75	15.53	81.0	314.14	94.16
S003	748,965,060	608,207,582	83.1	99.75	11.12	79.8	327.65	94.92

Supplementary Table S3 DNA methylation level of candidate genes[1]

Supplemental Table S3 DNA methylation level of candidate genes

Gene name	DNA methylation level	
	NOR	POI
IRF1	54.54	78.07
ZFR	27.68	59.58
PARP2	50.22	80.61
ULBP3	60.84	90.95
FABP3	17.39	49.89
MOB1A	43.56	78.07
RAB3C	57.9	94.23
PDE4D	57.9	93.23
LRP1B	46.87	88.9
ERC1	53.05	74.67
ATP8A2	39.26	61.48

[1] In order to calculate the methylation level of the sequence, we divided the sequence into multiple bins, with bin size is 10kb. The sum of methylated (reads(mC)) and unmethylated read counts(reads(C)) in each window were calculated. Methylation level (ML) for each window or C site shows the fraction of methylated Cs, and is defined as:

$$\mathbf{ML} = \frac{reads(mC)}{\mathrm{reads(mC)} + \mathrm{reads(C)}}$$

We modeled the sum mC of methylated counts as a binomial random variable with methylation rate r. Calculated ML was further corrected with the bisulfite non-conversion rate. Given the bisulfite non-conversion rate r, the corrected ML was estimated as:

$$\mathbf{ML(corrected)} = \frac{ML - r}{1 - \mathrm{r}}$$

Supplementary Table S4 Identification of related proteins by MS/MS

Supplemental Table S4 Identification of related proteins by MS/MS

Accession	Description	Area	Peptides
NP_066964.1	X-ray repair cross-complementing protein 5[Homo sapiens]	9.11E+09	48
NP_001275905.1	X-ray repair cross-complementing protein 6 isoform 1[Homo sapiens]	8.61E+09	48
NP_001609.2	poly [ADP-ribose] polymerase 1[Homo sapiens]	7.10E+09	64
NP_059447.2	major vault protein isoform 1[Homo sapiens]	6.97E+09	52
XP_006713191.1	PREDICTED:inosine-5'-monophosphate dehydrogenase 2 isoform X1[Homo sapiens]	5.65E+09	27
NP_002936.1	replication protein A 70 kDa DNA-binding subunit isoform 1[Homo sapiens]	3.54E+09	30
NP_001243439.1	single-stranded DNA-binding protein, mitochondrial precursor[Homo sapiens]	2.00E+09	7
NP_821133.1	tubulin beta chain isoform b[Homo sapiens]	1.22E+09	19
NP_002017.1	fibronectin isoform 3 preproprotein[Homo sapiens]	1.12E+09	52
NP_005900.2	microtubule-associated protein 1B isoform 1[Homo sapiens]	1.07E+09	48
NP_001138610.1	transcription factor p65 isoform 2 [Homo sapiens]	9.51E+08	18
NP_039269.2	DNA ligase 3 isoform alpha precursor protein-glutamine[Homo sapiens]	7.90E+08	34
NP_001310245.1	gamma-glutamyltransferase 2 isoform a[Homo sapiens]	5.79E+08	20
NP_004619.3	DNA repair protein complementing XP-C cells[Homo sapiens]	5.29E+08	29
NP_001349801.1	peroxisome proliferator-activated receptor alpha [Homo sapiens]	5.09E+08	18
NP_001284487.1	replication protein A 32 kDa subunit isoform 3[Homo sapiens]	4.50E+08	8
XP_006722822.2	PREDICTED: nuclear factor 1 C-type isoform X2[Homo sapiens]	4.30E+08	16
NP_150091.2	PC4 and SFRS1-interacting protein isoform 2[Homo sapiens]	3.68E+08	14
NP_005644.2	alpha-globin transcription factor CP2 isoform 1[Homo sapiens]	3.39E+08	12
XP_005251524.1	PREDICTED: nuclear factor 1 B-type isoform X1[Homo sapiens]	3.15E+08	10
NP_000468.1	serum albumin preproprotein [Homo sapiens]	3.10E+08	6
NP_055332.3	upstream-binding protein 1 isoform LBP-1b [Homo sapiens]	3.10E+08	12
NP_005345.3	transcription factor jun-D isoform JunD-FL [Homo sapiens]	3.10E+08	6
XP_005250918.1	PREDICTED: polyadenylate-binding protein 1 isoform X1 [Homo sapiens]	2.90E+08	22
NP_079031.2	SAFB-like transcription modulator isoform a [Homo sapiens]	2.90E+08	14
NP_001158884.1	nuclear factor NF-kappa-B p105 subunit isoform 2 proprotein [Homo sapiens]	2.90E+08	19
XP_011533233.1	PREDICTED: poly [ADP-ribose] polymerase 4 isoform X1 [Homo sapiens]	2.80E+08	33
NP_000874.2	inosine-5'-monophosphate dehydrogenase 1 isoform a [Homo sapiens]	2.60E+08	17
NP_055464.1	scaffold attachment factor B2 [Homo sapiens]	2.60E+08	22
NP_003394.1	transcriptional repressor protein YY1 [Homo sapiens]	2.60E+08	8
NP_065390.1	NF-kappa-B inhibitor alpha	2.20E+08	13
NP_006550.1	KH domain-containing, RNA-binding, signal transduction-associated protein 1 isoform 1 [Homo sapiens]	2.50E+08	9
NP_006657.1	ruvB-like 2 isoform 1 [Homo sapiens]	2.40E+08	19
NP_004441.1	enhancer of rudimentary homolog [Homo sapiens]	2.40E+08	1
NP_001193943.1	serpin H1 precursor [Homo sapiens]	2.20E+08	16
NP_054721.1	Inhibitor of nuclear factor kappa-B kinase subunit epsilon isoform 1[Homo	2.20E+08	13

	sapiens]		
NP_001395.1	elongation factor 1-gamma [Homo sapiens]	2.20E+08	12
NP_000414.2	keratin, type II cytoskeletal 2 epidermal [Homo sapiens]	2.20E+08	21
NP_001073027.1	heterogeneous nuclear ribonucleoprotein U-like protein 2 [Homo sapiens]	2.10E+08	15
NP_001410.2	ELAV-like protein 1 [Homo sapiens]	2.10E+08	10
NP_604391.1	cyclic AMP-responsive element-binding protein 1 isoform B [Homo sapiens]	2.10E+08	5
NP_006319.1	RNA-binding protein 14 isoform 1 [Homo sapiens]	2.10E+08	13
XP_016882687.1	PREDICTED: ubiquitin-60S ribosomal protein L40 isoform X1 [Homo sapiens]	2.10E+08	3
NP_002865.1	UV excision repair protein RAD23 homolog B isoform 1 [Homo sapiens]	2.00E+08	12
NP_002938.1	replication protein A 14 kDa subunit [Homo sapiens]	2.00E+08	5
XP_005249096.1	PREDICTED: histone H2B type 1-D isoform X1 [Homo sapiens]	2.00E+08	5
NP_005564.1	lamin-B1 isoform 1 [Homo sapiens]	1.90E+08	22
NP_954657.1	keratin, type I cytoskeletal 18 [Homo sapiens]	1.90E+08	11
NP_001531.1	heat shock protein beta-1 [Homo sapiens]	1.90E+08	8
NP_000084.3	collagen alpha-1(V) chain isoform 1 preproprotein [Homo sapiens]	1.80E+08	22
NP_002941.1	dolichyl-diphosphooligosaccharide--protein glycosyltransferase subunit 1 precursor [Homo sapiens]	1.80E+08	19
XP_006713210.1	PREDICTED: microtubule-associated protein 4 isoform X5 [Homo sapiens]	1.80E+08	16
NP_001305116.1	heterogeneous nuclear ribonucleoprotein K isoform d [Homo sapiens]	1.80E+08	11
NP_001311233.1	dolichyl-diphosphooligosaccharide--protein glycosyltransferase subunit 2 isoform 7 precursor [Homo sapiens]	1.80E+08	13
NP_000081.1	collagen alpha-1(III) chain preproprotein [Homo sapiens]	1.80E+08	19
NP_003277.1	DNA topoisomerase 1 [Homo sapiens]	1.80E+08	16
NP_001347.3	ATP-dependent RNA helicase DDX3X isoform 1 [Homo sapiens]	1.60E+08	14
NP_009185.2	bifunctional polynucleotide phosphatase/kinase [Homo sapiens]	1.60E+08	15
NP_001104026.1	filamin-A isoform 2 [Homo sapiens]	1.60E+08	57
XP_005249786.1	PREDICTED: heterogeneous nuclear ribonucleoproteins A2/B1 isoform X1 [Homo sapiens]	1.60E+08	14
NP_000098.1	DNA damage-binding protein 2 isoform WT [Homo sapiens]	1.60E+08	12
NP_002425.2	DNA-3-methyladenine glycosylase isoform a [Homo sapiens]	1.60E+08	11
XP_016881278.1	PREDICTED: ATP synthase subunit alpha, mitochondrial isoform X1 [Homo sapiens]	1.60E+08	12
NP_001070910.1	heterogeneous nuclear ribonucleoproteins C1/C2 isoform a [Homo sapiens]	1.60E+08	8
NP_002219.1	transcription factor AP-1 [Homo sapiens]	1.60E+08	7
NP_005207.2	dolichyl-diphosphooligosaccharide--protein glycosyltransferase 48 kDa subunit precursor [Homo sapiens]	1.60E+08	8
NP_000959.2	60S ribosomal protein L4 [Homo sapiens]	1.50E+08	10
NP_002408.3	proliferation marker protein Ki-67 isoform 1 [Homo sapiens]	1.50E+08	32
XP_016882252.1	PREDICTED: interleukin enhancer-binding factor 3 isoform X1 [Homo sapiens]	1.50E+08	15
NP_005262.1	glutamate dehydrogenase 1, mitochondrial isoform a precursor [Homo sapiens]	1.50E+08	12
NP_005244.1	fos-related antigen 2 [Homo sapiens]	1.50E+08	8
NP_001182177.1	aprataxin isoform e [Homo sapiens]	1.50E+08	10
NP_066299.2	myosin light polypeptide 6 isoform 1 [Homo sapiens]	1.50E+08	6

NP_005959.2	heterogeneous nuclear ribonucleoprotein M isoform a [Homo sapiens]	1.40E+08	22
XP_011519222.1	PREDICTED: zinc finger protein 384 isoform X1 [Homo sapiens]	1.40E+08	5
XP_011512327.2	PREDICTED: ankycorbin isoform X5 [Homo sapiens]	1.40E+08	26
NP_001524.2	heterogeneous nuclear ribonucleoprotein L isoform a [Homo sapiens]	1.40E+08	8
NP_002130.2	RNA-binding motif protein, X chromosome isoform 1 [Homo sapiens]	1.40E+08	6
XP_005248694.1	PREDICTED: heterogeneous nuclear ribonucleoprotein Q isoform X3 [Homo sapiens]	1.40E+08	14
NP_001258898.1	heat shock protein HSP 90-beta isoform a [Homo sapiens]	1.30E+08	18
NP_005475.2	poly [ADP-ribose] polymerase 2 isoform 1 [Homo sapiens]	1.30E+08	10
NP_002152.2	isoleucine--tRNA ligase, cytoplasmic [Homo sapiens]	1.30E+08	23
NP_009210.1	splicing factor U2AF 65 kDa subunit isoform a [Homo sapiens]	1.30E+08	7
NP_004506.2	interleukin enhancer-binding factor 2 isoform 1 [Homo sapiens]	1.30E+08	7
XP_016868908.1	PREDICTED: aspartyl/asparaginyl beta-hydroxylase isoform X1 [Homo sapiens]	1.30E+08	10
NP_060979.2	leucine-rich repeat-containing protein 59 [Homo sapiens]	1.30E+08	7
NP_001950.1	elongation factor 1-beta [Homo sapiens]	1.30E+08	3
XP_011539528.1	PREDICTED: D-3-phosphoglycerate dehydrogenase isoform X1 [Homo sapiens]	1.30E+08	9
NP_005879.1	phosphate carrier protein, mitochondrial isoform a precursor [Homo sapiens]	1.20E+08	6
NP_002430.3	DNA mismatch repair protein Msh3 [Homo sapiens]	1.20E+08	23
NP_000080.2	collagen alpha-2(I) chain precursor [Homo sapiens]	1.20E+08	15
NP_001104547.1	ezrin [Homo sapiens]	1.20E+08	20
NP_000997.1	40S ribosomal protein S3a isoform 1 [Homo sapiens]	1.20E+08	9
NP_006796.1	heterogeneous nuclear ribonucleoprotein A0 [Homo sapiens]	1.20E+08	5
NP_001518.3	histone deacetylase 2 [Homo sapiens]	1.20E+08	9
NP_004437.2	bifunctional glutamate/proline--tRNA ligase [Homo sapiens]	1.10E+08	25
XP_006723632.1	PREDICTED: 1-phosphatidylinositol 4,5-bisphosphate phosphodiesterase beta-4 isoform X1 [Homo sapiens]	1.10E+08	23
NP_001009999.1	lysine-specific histone demethylase 1A isoform a [Homo sapiens]	1.10E+08	16
NP_001307526.1	probable ATP-dependent RNA helicase DDX5 isoform b [Homo sapiens]	1.10E+08	16
XP_016868895.1	PREDICTED: RNA-binding protein 12B isoform X1 [Homo sapiens]	1.10E+08	18
XP_005268883.1	PREDICTED: heterogeneous nuclear ribonucleoprotein A1 isoform X1 [Homo sapiens]	1.10E+08	10
NP_065393.1	reticulon-4 isoform A [Homo sapiens]	1.10E+08	5
NP_001677.2	ATP synthase subunit beta, mitochondrial precursor [Homo sapiens]	1.10E+08	10
NP_001406.1	eukaryotic translation initiation factor 2 subunit 3 [Homo sapiens]	1.10E+08	15

Supplementary Table S5 POI and NOR patients' clinic information

Supplemental Table S5 POI and NOR patients' clinic information

Group	Number	Age	Systolic Pressure (mmHg)	Diastolic Pressure (mmHg)	BMI	FSH (IU/L)	LH (IU/L)	PRL (ng/ml)	T (ng/ml)	E2 (pg/ml)	TSH (uIU/ml)	Ultrasonography (numbers of unilateral ovaries)
POI	1	30	105	72	21.6	27.12	7.45	10.13	0.93	16.3	1.94	1 follicles
	2	22	107	80	21	26.28	8.27	6.22	0.65	19.3	2.25	1 follicles
	3	26	119	80	20.3	27.66	9.47	7.59	0.52	15.5	1.85	1 follicles
	4	28	112	64	18.6	24.39	8.87	11.5	0.91	11.54	2.3	1 follicles
	5	29	102	70	23.3	26.19	9.63	8.76	0.49	15.2	1.56	1 follicles
	6	29	112	80	22.5	23.61	7.68	23.66	0.76	18.7	2.01	1 follicles
	7	30	114	76	18.8	23.23	7.79	28.29	0.33	24.35	1.25	1 follicles
	8	26	118	69	20	24.79	8.29	13.24	0.39	26.31	2.13	1 follicles
	9	27	120	83	23.2	28.22	9.28	19.22	0.81	19.11	1.98	1 follicles
	10	25	110	70	18.2	27.58	9.1	18.36	0.21	17.12	1.77	1 follicles
NOR	1	25	113	74	22.6	4.28	3.41	5.62	0.33	66.47	1.63	7 follicles
	2	27	112	81	20.7	6.57	3.91	7.02	0.28	46.56	1.35	8 follicles
	3	28	108	70	22.2	5.09	3.09	10.82	0.2	58.75	1.96	6 follicles
	4	28	112	65	20.9	8.39	7.3	11.46	0.32	33.76	2.45	6 follicles
	5	24	107	71	29.4	6.79	1.93	16.6	0.26	56.48	1.66	7 follicles
	6	23	104	69	18.6	5.49	1.35	11.6	0.52	49.19	2.12	6 follicles
	7	26	111	75	27.1	6.36	2.94	6.4	0.26	59.6	2.15	6 follicles
	8	29	118	64	22.5	7.58	5.99	22.91	0.47	39.6	2.17	7 follicles
	9	30	103	74	21.5	5.04	3.37	19.1	0.95	42.9	1.88	6 follicles
	10	25	102	82	19	6.81	6.24	25.83	0.35	52.91	1.45	6 follicles
	11	27	119	78	24.8	6.23	5.89	26.58	0.4	40.79	1.55	8 follicles
	12	28	115	74	27.9	5.6	8.86	6.32	0.44	48.15	1.3	8 follicles
	13	30	100	76	21.5	7.98	4.48	25.25	0.14	50.77	2.26	6 follicles
	14	24	105	83	22.5	8.58	5.37	15.3	0.28	56.9	2.33	7 follicles
	15	26	114	77	23.4	5.32	7.02	25.41	0.52	52.2	1.13	7 follicles
	16	27	113	79	19.1	6.37	5.92	25.66	0.27	45.06	1.36	6 follicles
	17	25	110	82	17.7	8.42	5.23	14.84	0.1	55.87	1.35	8 follicles
	18	23	112	74	22.7	5.48	4.14	14.81	0.5	50.6	1.45	7 follicles
	19	24	115	76	18.8	6.52	4.63	7.78	0.32	44.64	2.39	6 follicles

IV. Reference

Al Nakouzi, N., Wang, C.K., Beraldi, E., et al. (2016). Clusterin knockdown sensitizes prostate cancer cells to taxane by modulating mitosis. EMBO Mol Med *8*, 761-778.

European Society for Human, R., Embryology Guideline Group on, P.O.I., Webber, L., et al. (2016). ESHRE Guideline: management of women with premature ovarian insufficiency. Hum Reprod *31*, 926-937.

Lobb, D.K., and Younglai, E.V. (2006). A simplified method for preparing IVF granulosa cells for culture. J Assist Reprod Genet *23*, 93-95.

Morgan, S., Campbell, L., Allison, V., et al. (2015). Culture and co-culture of mouse ovaries and ovarian follicles. J Vis Exp.
